# Putting in the graft: cell wall remodeling genes underlie graft union development

**DOI:** 10.1093/plphys/kiag088

**Published:** 2026-02-26

**Authors:** Erin Cullen

**Affiliations:** Assistant Features Editor, Plant Physiology, American Society of Plant Biologists

Grafting, a technique where 2 different plants are combined to function as a single chimeric organism, has been practised for thousands of years. To perform a graft, the stem or shoot (termed a scion) is detached and attached to the roots (termed a rootstock) of a related plant. Grafting is a useful method that allows both hobbyists and commercial growers to combine the beneficial traits of 2 different plants. For example, rigorous rootstocks can be combined with a scion with excellent fruiting qualities.

For a successful graft, it is necessary for the vascular tissues of the 2 plants to fuse. After the graft is performed, the injured cells form a necrotic layer, which is then filled with a callus (a mass of proliferating cells) (Melnyk et al 2018). Cell wall remodeling then takes place to allow the reunification of the 2 halves (Cookson et al 2013). A homograft is when a scion and rootstock from the same species are grafted together, whereas a heterograft is when a scion and rootstock from 2 different species are grafted together. Grafting is commonly used in the commercial production of Cucurbitaceae and Solanaceae crops (Tsaballa et al 2021). Bottle gourd (*Lagenaria siceraria*) is often used as a Cucurbitaceae rootstock; however, it exhibits reduced compatibility with melon (*Cucumis melo*).

A recent study in *Plant Physiology* by Xiong et al (2026) investigated the physiological and molecular basis of reduced graft compatibility between melon scions and bottle gourd rootstocks. The authors tested the compatibility of grafts between 10 melon varieties and 9 bottle gourd varieties. The melon variety “Akekekouqi” (AK) was utilized as a scion and grafted onto the multiple bottle gourd varieties as rootstocks. Plants were shorter compared to controls. A similar trend was observed when 10 varieties of melon scions were grafted onto the bottle gourd “H23” as a rootstock. The authors examined the phenotype of heterograft plants in detail using the melon variety “AK” as a scion and bottle gourd variety “H23” as a rootstock. Heterografted plants exhibited an increase in interfascicular layers (vascular cambium arising between vascular bundles) in the scion. Unbalanced growth at the graft junction may explain the significant reduction in plant height and root growth in heterografted plants.

Vascular reconnection in heterografts was examined using scion wilting rates as a proxy for functional recovery of xylem transport. Xylem reconnection was established for both compatible melon/melon homografts and less compatible melon/bottle gourd heterografts, although reconnection was slower in less compatible grafts. The authors then assessed phloem status using the symplastic tracer 5,6-carboxyfluorescein (Fig. 1). Phloem reconnection was observed, but reconnection was slower in melon/bottle gourd heterografts, possibly due to increased callose deposition in heterografts. Phloem transport efficiency was also significantly reduced in heterografts. Interestingly, more starch accumulation was observed at heterograft junctions, and sections revealed higher levels of callus proliferation in heterografts. Therefore, lower phloem connectivity in heterografts may lead to more starch accumulation, increasing callus proliferation.

**Figure 1:**
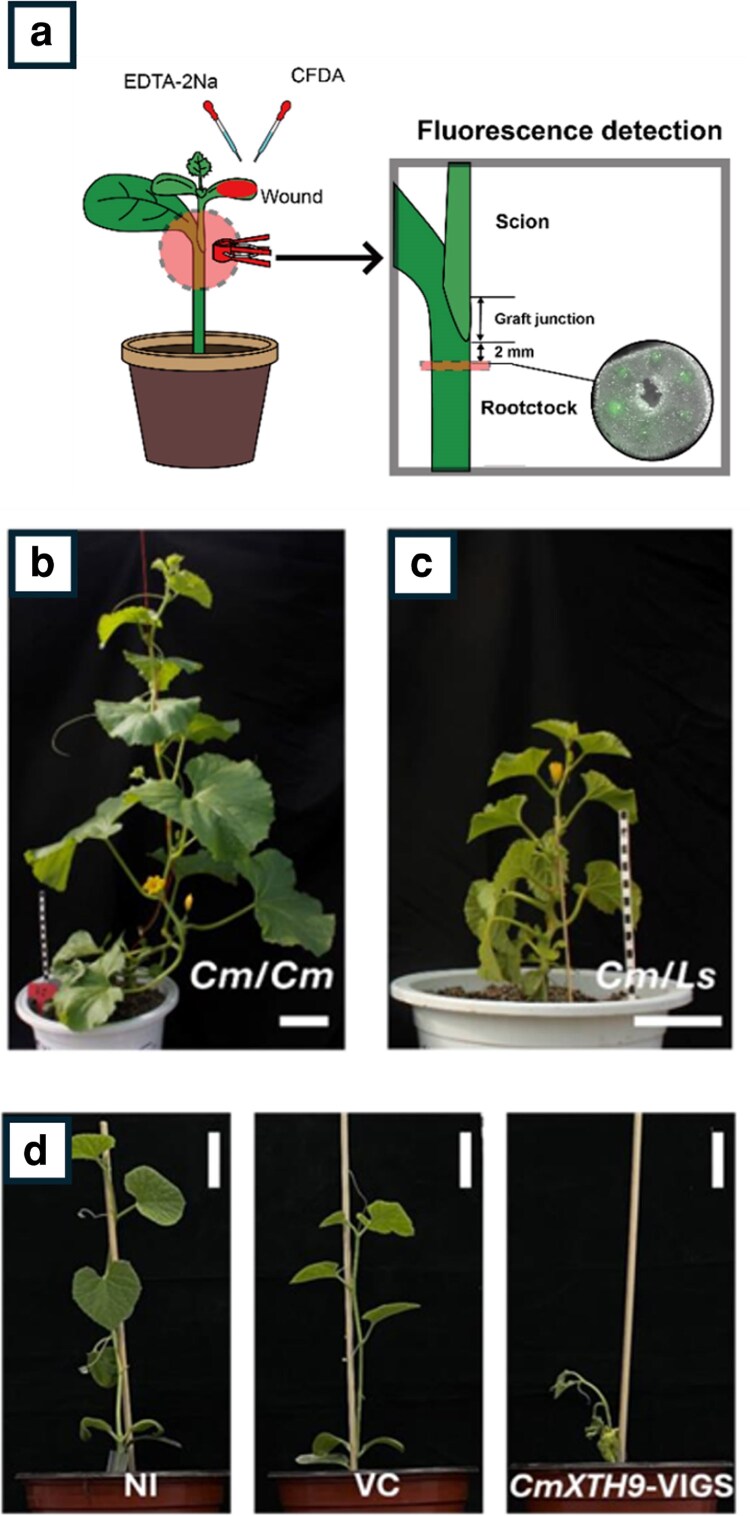
Melon/bottle gourd heterografts exhibit lower compatibility than melon/melon homografts, and *XTH9* is essential for grafting success in melon/bottle gourd grafts (adapted from Figs. 1 and 4 and supplementary Fig. S6 from Xiong et al (2026)). **a)** Schematic of phloem connectivity experiments used in Xiong et al (2026). The scion, rootstock, and graft junction are labeled. Fluorescence is detected in the rootstock after symplastic tracer is applied to the scion. **b)** The typical phenotype of a melon/melon homograft. **c)** The typical shorter phenotype of a melon/bottle gourd heterograft. **d)** Representative melon/bottle gourd heterografts 28 days after grafting in VIGS experiments, in which infected melon scions were grafted onto bottle gourd rootstocks. *CmXTH9*-VIGS, partial sequence of CmXTH9 for gene silencing; NI, non-infected; VC, empty vector control.

To interrogate the molecular mechanisms underlying vascular connection and connectivity at the graft junction, the authors performed RNAseq, dissecting the scion section of the graft junction at multiple timepoints. A gene ontology enrichment analysis on the differentially expressed genes in the heterografts compared to homografts revealed that xyloglucan metabolic process was the most enriched term, seconded by the hydrogen peroxide metabolic process. Therefore modification of xyloglucan (and increased levels of hydrogen peroxide) may be typical at the graft junction. The gene *xyloglucan endotransglucosylase9* (*XTH9*) was identified as a candidate gene for further analyses. Notably, xyloglucan endotransglucosylase genes encode enzymes that modify xyloglucan, an important component of the primary cell wall in many plants (Eklöf and Brumer 2010; Kim et al 2020).

To understand the function of *XTH9* in melon, virus-induced gene silencing (VIGS) was used to generate *CmXTH9*-VIGS plants. *CmXTH9*-VIGS plants exhibited reduced height, and the heterograft success rate was significantly reduced. At the graft junction of infected plants, necrotic layer remnants and reduced callus formation were observed, along with a strong upregulation of *XTH* genes in heterografts. Interestingly, in less compatible grafts there was stronger upregulation of *XTH* genes. The authors also examined *XTH* gene family expression in multiple species. They found significant upregulation of *XTH* genes after grafting in diverse species, such as *Arabidopsis thaliana*, *Nicotiana benthamiana*, and *Oryza sativa*. This may suggest that *XTH* gene function is conserved across species.

The function of *XTH4* and *XTH7* genes in *A. thaliana* was explored (corresponding to *CmXTH9*) to investigate the role of *XTH* genes in other species. Transcriptional reporters revealed expression in both the proliferating callus and vascular tissues of the graft junction, particularly in the scion. Interestingly, no phenotype was observed when grafting single *Atxth4* and *Atxdh7* T-DNA mutants; however, there was a significant reduction in grafting success in double mutants. Thus, *XTH* genes may show functional redundancy.

In conclusion, this study helps to illuminate the molecular basis of graft compatibility. Cell proliferation at the graft junction, promoted by upregulation of *XTH* gene family members, may act to generate a physical center, creating a functional graft junction. This study provides useful information that can inform breeding strategies aiming to improve graft success between species. In the future, it would be interesting to investigate the role of additional enzymes involved in cell wall remodeling to understand the full gene network underlying graft union development.

## Data Availability

No new data were generated or analysed in support of this research.
